# NERI: network-medicine based integrative approach for disease gene prioritization by relative importance

**DOI:** 10.1186/1471-2105-16-S19-S9

**Published:** 2015-12-16

**Authors:** Sérgio N Simões, David C Martins, Carlos AB Pereira, Ronaldo F Hashimoto, Helena Brentani

**Affiliations:** 1 Institute of Mathematics and Statistics - University of São Paulo, São Paulo-SP, Brazil; 2 Federal Institute of Espírito Santo, Serra-ES, Brazil; 3 Center of Mathematics, Computation and Cognition - Federal University of ABC, Santo André-SP, Brazil; 4 Department of Psychiatry - University of Sao Paulo Medical School, São Paulo-SP, Brazil; 5 Research Center on Neurodevelopment and Mental Health - University of Sao Paulo, São Paulo-SP, Brazil; 6 National Institute of Developmental Psychiatry for Children and Adolescents (INCT-CNPq), Sĕo Paulo-SP, Brazil

**Keywords:** Network-Medicine, relative importance, gene prioritization, complex diseases, PPI network

## Abstract

**Background:**

Complex diseases are characterized as being polygenic and multifactorial, so this poses a challenge regarding the search for genes related to them. With the advent of high-throughput technologies for genome sequencing, gene expression measurements (transcriptome), and protein-protein interactions, complex diseases have been sistematically investigated. Particularly, Protein-Protein Interaction (PPI) networks have been used to prioritize genes related to complex diseases according to its topological features. However, PPI networks are affected by ascertainment bias, in which more studied proteins tend to have more connections, degrading the results quality. Additionally, methods using only PPI networks can provide only static and non-specific results, since the topologies of these networks are not specific of a given disease.

**Results:**

The goal of this work is to develop a methodology that integrates PPI networks with disease specific data sources, such as GWAS and gene expression, to find genes more specific of a given complex disease. After the integration of PPI networks and gene expression data, the resulting network is used to connect genes related to the disease through the shortest paths that have the greatest concordance between their gene expressions. Both case and control expression data are used separately and, at the end, the most altered genes between the two conditions are selected. To evaluate the method, schizophrenia was adopted as case study.

**Conclusion:**

Results show that the proposed method successfully retrieves differentially coexpressed genes in two conditions, while avoiding the bias from literature. Moreover we were able to achieve a greater concordance in the selection of important genes from different microarray studies of the same disease and to produce a more specific gene set related to the studied disease.

## 

Prioritization of relevant genes associated with complex diseases is a significant challenge, because such diseases are polygenic and multifactorial. In addition, patients with the same complex disease can present different genetic perturbations [1]. With the advent of high-throughput technologies for genome sequencing, gene expression measurements (transcriptome), and protein-protein interactions mapping, complex diseases have been sistematically investigated. Genomewide association studies (GWAS) is an approach that has improved our comprehension of the genetic basis of many complex traits [2]. However, it fails in revealing the relatively small effect sizes found in most genetic variants [3,4]. Other studies suggest that combining GWAS approach with transcriptome analyses, such as eQTL mapping, reduces the number of false positives and helps the discovery of new functional loci [5,6].

Protein-protein interaction (PPI) networks have also become an important tool to study the complex molecular relationships in a living organism. Barabási *et al *[7] summarized a series of hypotheses and principles (*Network Medicine Hypotheses*) which link topological properties of PPI networks to biological functionalities. Some of these hypotheses are often used to prioritize candidate genes related to a given disease. We highlight three hypotheses:

• *disease module hypothesis*: genes products associated to the same disease phenotype tend to form a cluster in the PPI network;

• *network parsimony*: shortest paths between known disease genes often coincide with disease pathways;

• *local hypothesis*: gene products associated with similar diseases are likely to strongly interact with each other.

In fact, many recent works have analyzed topological properties of PPI networks to comprehend genetic diseases assuming the Network Medicine hypotheses [7-10]. Such methods take as input a PPI network, a set of seeds (usually originated from GWAS) and candidate genes and output a set of candidates ranked by a score. They can be categorized in two main approaches: (i) local (e.g. direct neighbors and shortest paths - [11]) and (ii) global (e.g random walk with restart [8,9,12] and network propagation [10]). Kohler *et al *[8] proposed to use random walks with restart (RWR) method to prioritize candidate disease genes. Vanunu *et al *[10] proposed to use network propagation method, a slight variation of RWR.

Nevertheless, PPI networks data are noisy and incomplete [13,14], which impact the accuracy of the candidate gene prioritization methods. Besides, largely studied proteins tend to be highly connected, which are favored by methods relying on topological properties assessment. Thus, due to this issue - usually called "ascertainment bias" - genes that are not well studied, hardly appear among the top ranked ones [15]. Erten *et al *[15] proposed statistical adjustments to the RWR method in order to address the 'ascertainment bias', in which well studied genes tend to be favored by methods based on PPI just because of their high connectivity degree. This method intends to detect both high and low degree related genes instead of only the higher degree genes in the seeds neighborhood. However, like other aforementioned methods, it is based solely on the static PPI network and seed proteins information.

To properly understand the mechanisms underlying complex diseases, techniques involving integrative analysis of data originated from many sources have been developed. There is a lot of genomic, transcriptomic and proteomic data available from different complex diseases in public databases. To improve the identification and prioritization of genes associated with complex diseases, some works began to integrate PPI networks with information derived from other omics data, which have contributed to a better understanding of gene functions, interactions, and pathways [16,17]. The integration of PPI networks and gene expression data has improved disease classification and identification of disease specific deregulated pathways [9,16-20]. The reader is referred to [21] for a survey of these integrative approaches. However, the resulting gene lists from different studies almost does not present overlap.

In this paper, we proposed a methodology that integrates data from association studies (GWAS), gene expression profiles and PPI datasets. Then, by assuming the Network Medicine hypotheses, given a set of seed genes (obtained by GWAS) the method analyzes its neighborhood by retrieving all shortest paths between them and their direct neighbors. Next, the method selects those shortest paths that present the largest gene expression concordance among the genes in the paths. This is performed separately in two conditions (case and control) generating two networks, in which each gene (and interaction) is scored taking into account its (a) distance from seeds; (b) the gene expression concordance (of the genes in the paths to which it belongs); and (c) its frequency across the paths. Finally the method analyzes the differences between the networks under two conditions to select the most altered genes (and interactions) according to the score aforementioned.

There is an increasing usage of systems biology approaches in psychiatric diseases as they are considered complex diseases. In the psychiatric field there are databases of autism [22,23], schizophrenia [24] and Alzheimer's disease [25] trying to integrate information from different sources of studies: GWAS, transcriptome and Interactome, etc. To validate our method we adopted schizophrenia data as case study. Schizophrenia is a major psychiatric disease affecting ~1% of the world population. It is commonly considered a complex disease with multiple genetic and environmental factors involved. However, genetic factors impact substantially on the risk of developing the disease, with an estimated heritability of ~80% [26]. Jia *et al *[27] used data from different schizophrenia studies: Association, Linkage, Expression, Literature search, Gene Ontology and Gene Network. For most data categories, gene score was calculated based on P-values, while for literature search they assigned score for a gene based on the number of keywords being hit in the search. Similarly, a score was assigned to a gene based on the number of neuro-related GO terms annotated to the gene. Then the genes were ranked based on the weighted average of such scores. They verified that several previous works found genes related to schizophrenia, but the overlap among those sets of genes is very small. The overlap of genes coming from transcriptome analysis compared to genes coming from genome-wide association studies (GWAS) are almost null. Therefore, integration of different datasources is still an open problem in the psychobiology of schizophrenia. By applying our method to 3 different gene expression schizophrenia studies, it successfully retrieved differentially coexpressed genes in two conditions, while avoiding the ascertainment bias. Additionally, the proposed method could obtain a greater replicability in the selection of important genes from different microarray studies of the same disease and to produce a more specific gene set related to the studied disease.

## Materials and methods

### Materials

The PPI network was integrated as described in the section Data integration. We used 3 different PPI datasets: (i) HPRD (Human Protein Reference Database) [28]; (ii) IntAct (IntAct Molecular Interaction Database) [29]; (iii) MINT (Molecular Interaction database) [30]. The gene expression datasets were obtained from SNCID (Stanley Neuropathology Consortium Integrative Database) [24]. In SNCID there exist several neuropathological datasets, from which three schizophrenia gene expression database were selected for the experiments: KATO (35 control and 34 disease samples), ALTARC (33 control and 34 disease samples) and BAHN (29 control and 21 disease samples). The normalization of these datasets was performed by Affymetrix package using RMA [31,32] in R language.

We adopted as seeds (disease-related genes) a set of 38 genes (called 'core genes') obtained from the SZGR database [27], since such genes presented significant meta-analyses results from disease association studies. Among these 'core genes', 30 were present in the main connected component of the composed PPI network.

### Data integration

Since there are several human PPI network datasets available, some of these network datasets are combined to obtain a unique PPI network. Then, proteins and transcripts are mapped to their corresponding coding genes (gene symbols). Since more than one transcript can be mapped to the same gene, the gene expression profile is represented by the median of its transcripts expression. In this way, we compose the PPI network by integrating it with the gene expression data - keeping only genes that belong to both. From now on, in the context of PPI networks, we will use the term 'gene' referring to their respective proteins.

### Feature extraction

By assuming local, disease module and parsimony principle hipotheses, this step extracts features of the genes in the neighborhood of the seeds aiming to discover genes potentially related to the disease in study. Following the local hypothesis, the set of seeds *S *is grown to include their neighbors *N *(direct interactions), obtaining a new set *S *∪ *N*. Then, by following the network parsimony principle, the two sets *S *and *S *∪ *N *are connected by shortest paths, leading to a subnetwork representing the neighborhood of the seed genes. All genes in this neighborhood are considered candidates to be related to the disease.

#### Shortest paths selection

According to the disease module hypothesis, genes related to the same disease tend to compose modules in regions of PPI network. Several works use the shortest path algorithm to infer biological pathways and/or subnetworks with groups of genes related to the disease, showing that many intermediate genes are also related to the disease [33-38].

Between a pair of genes in the human PPI network there may be several possible shortest paths, and some of these paths can be much more valuable than others from the biological perspective. Assuming that genes more correlated with the seed genes tend to be associated with the disease [39], a criterion that evaluates a given shortest path based on the coexpression of its genes among them and the seeds is desirable. In this way, we adopted a modified version of Kendall's Concordance Coefficient (*W_max_*) to measure the overall expression concordance among the genes of a given path [40,41] (see Kendall's Concordance Coefficient next).

When selecting the best shortest path for a given pair of genes, the problem of ties arise, where multiple shortest paths might have the same (or almost the same) *W*_*max *_value. Thus, to avoid losing important information about biologically relevant shortest paths, a factor *ε *is defined to include all paths *P_i _*which present concordance value *W_max_*(*P_i_*) ≥ *W_max_*(P*)(1 - *ε*), where P^* ^is the path with the best concordance value for a given pair of genes. For instance, suppose that *W_max_*(P*) = 0, 6 and *ε *= 0, 05 for a given gene pair. Then all shortest paths presenting *W_max _*≥ 0.6 × (1 - 0.05) = 0.57 are considered tied with the best one and thus included as the best shortest paths for the considered gene pair.

#### Kendall's Concordance Coefficient

To compute the Kendall's Concordance Coefficient *W *value independently of genes being up or down-regulated, we did an adaptation on the computation of this coefficient, in which all possible ranking inversions are considered. The ranking inversion which maximizes the concordance *W *among the genes is chosen, resulting in the modified value *W_max _*for a given path - this is the criterion adopted to evaluate the coexpression of a path. This criterion is taken into account to select the best shortest paths for a given gene pair as aforementioned.

#### Genes scoring in a given condition

Inspired by several works presenting different ways to prioritize genes in a PPI network [11,27,42], we derived a score to assess the relative importance of a given gene considering topological and transcriptional aspects in a given condition (disease or control subnetworks) for genes belonging to the shortest paths selected in the previous step. Regarding the topological aspects, we use concepts similar to closeness and betweenness. Concerning the transcriptional aspects, we use *W_max _*value to measure how genes from a given path are coexpressed. Considering all these aspects, we proposed the formula given by Equation 1 to score the relative importance of a gene related to the seeds and related to a given condition:

(1) $$\sigma \left(g\right)=\underset{\begin{array}{c} s\in S\\ t\in S\cup N\\ P_{st}\in P_{st}^{*} \end{array}}{\sum }\lambda^{-d_{sg}}\times W_{max}\left(P_{st}\right)\times 1_{g\in P_{st}}$$

where *d_sg _*is the distance between nodes *s *and *g *in the PPI, $P_{st}^{*}$ is the set of selected shortest paths from *s *to *t*, and $1_{g\in P_{st}}$ is the indicator function which returns 1 if *g *belongs to *P_st _*and 0 otherwise.

The first term $\left(\lambda^{-d_{sg}}\right)$, similar to closeness, seeks to penalize the distance between a given gene *g *and a seed *s*. The greater the distance to *s *the lower the importance of the gene *g*. The second term (*W_max_*) seeks to measure the concordance of gene expression profiles of the selected shortest paths containing gene *g*. Thus, this term seeks to benefit genes belonging to highly correlated shortest paths in terms of gene expression. The last term ($1_{g\in P_{st}}$) just means that the score of a gene *g *will be increased according to the multiplication of the first two terms if *g *belongs to *P_st_*. This equation assumes additivity of seeds contribution to the score of a given gene. It is applied to control and disease sub-networks to compare the scores of a given gene in two conditions (resulting in two gene score values *σ_C _*(*g*) and *σ_D _*(*g*), for control and disease conditions respectively).

### Gene selection

The problem of classifying genes as differentially altered resembles to identification of differentially expressed genes in microarray experiments. Among the methods proposed in literature for this purpose, we highlight the parametric method MAID [43]. Based on this method, we propose some alterations which use scores *σ_C _*and *σ_D _*to obtain the most altered genes in two conditions. For this, our proposed method performs two similar transformations to those applied by MA-plot method [44] - one regarding the intensity *A *and another regarding the alterations *M*. The intensity value (*x*-axis) of a given gene *g *is determined by Equation 2:

(2) $$X\left(g\right)=\sigma_{C}\left(g\right)+\sigma_{D}\left(g\right)$$

Besides, the alteration score value (*y*-axis) to compare of a given gene *g *is defined by Equation 3:

(3) $$\Delta \left(g\right)=\frac{\sigma_{D}\left(g\right)-\sigma_{C}\left(g\right)}{\sigma_{C}\left(g\right)+\sigma_{D}\left(g\right)}=\frac{\sigma_{D}\left(g\right)-\sigma_{C}\left(g\right)}{X\left(g\right)}$$

The alteration score Δseeks to measure the relative difference of the scores *σ*_*C*_(*g*) and *σ*_*D*_(*g*) between two conditions (control and disease). It varies from -1 to +1, where the negative values mean the control score is larger than disease score and the positive means the opposite. Values close to zero mean almost null difference between *σ*_*C*_(*g*) and *σ*_*D*_(*g*). When assessing relatives differences, in principle genes with larger absolute Δ(*g*) values would be more altered, but large values in denominator (*X*(*g*) = *σ*_*C*_(*g*) + *σ*_*D*_(*g*)) present smaller variations in the Δ(*g*) score - which is a similar problem found in classifying differentially expressed genes by using MA-plot (where large values of *A *present smaller variations in *M*). Indeed, genes with high (*X*(*g*) = *σ*_*C*_(*g*) + *σ*_*D*_(*g*)) values tend to present smaller Δ(*g*) scores. Hence, the idea is to differentially select genes according to their *X *values.

To select the most altered genes, we adapt the parametric method MAID (MA-plot-based signal intensitydependent fold-change criterion) [43], but instead of applying exponential approximation (*f *(*x*) = *ae^-bx^*) to fit the points in the interquartile range, we use the power-law approximation (*f *(*x*) = *ax^-k^*) - since it decays smoother. The curve fits the third quartile of Δ values in each interval considered in the domain *X *(such intervals are defined by a sliding window of a given length *W_len _*and step size *W_step_*). Hence, to consider the *X*(*g*) effect, the idea is to normalize the alteration score Δ(*g*) by its respective *X*(*g*) value according to Equation 4.

(4) $$\Delta \prime \left(g\right)=\frac{\Delta \left(g\right)}{f\left(X\left(g\right)\right)}=\frac{\sigma_{D}\left(g\right)-\sigma_{C}\left(g\right)}{X\left(g\right)f\left(X\left(g\right)\right)}$$

In this way, small values of *X *(which tend to present larger variations of alterations) are divided by a larger denominator, and as *X *increases (and tend to present smaller alteration values), such denominator decreases to compensates the effect of smaller alterations presented by larger *X*. The Δ*'*(*g*) signal indicates in which condition a given gene *g *has a larger score. For ranking purposes, the larger the abolute value |Δ*'*(*g*)|, the better the gene *g*.

## Results and discussion

**Figure 1:**
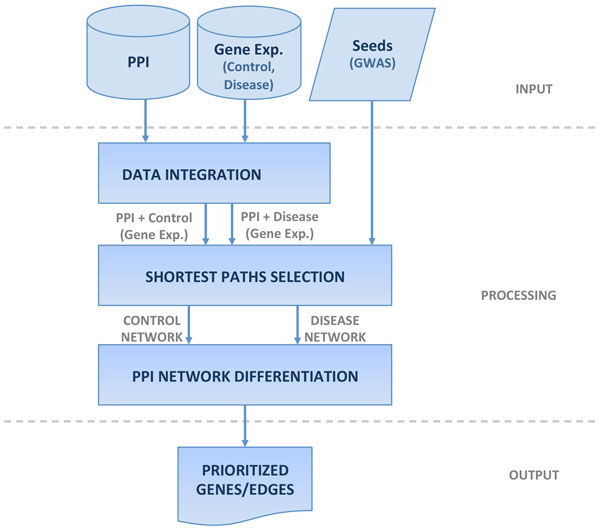
**Overview of the proposed method**.

In our approach (see Figure 1) we use as input: PPI network data, gene expression data and a set of seed genes related to our case study (schizophrenia). The gene expression profiles were obtained from two conditions: control and disease, and were assigned to the network nodes (genes) according to the adopted database (KATO, ALTARC and BAHN studies).

After the integration of PPI and gene expression datasets, the resulting network contained 9,554 nodes and 61,998 interactions.

### Parameters setup

From the differential analysis of the PPI network, some parameters need to be defined. Table 1 presents the list of parameters and its respective values adopted in the experiments.

**Table 1:** Parameter values adopted in the experiments.

Parameter	Value	Description
*W_len_*	2	Sliding window length. Such window defines the intervals in which the points will be considered to calculate the interquartile range values (IQR).

*W_step_*	1	Sliding window step size.

*X_ini_*	5	Threshold for which only genes with *X *= (*σ_C _*+ *σ_D_*) larger than this value are considered in the analysis.

*ε*	0.05	Factor used to deal with ties.

### Methods comparison (DADA vs RWR vs *X *vs Δ*'*)

This section presents a comparative analysis involving DADA and RWR methods. Our proposed method outputs the scores *X *and Δ*'*, which are both involved in this analysis. To facilitate the explanation, from now on we call *X *and Δ*' *as "methods", even though they are only resulting values of the same method.

Given lists containing the top *N *genes ranked by each method, we analyze how many genes are in the intersection between such rankings and how much similar are the ranking orders of the genes in common. We also compare the top *N *rankings of the four methods with the ranking obtained by gene/protein degree in decreasing order (the degree of a given protein is its number of interactions in the PPI network). We use expression data from KATO study in these analyses, since similar results were obtained by using expression data from ALTARC and BAHN studies (results not shown).

**Figure 2:**
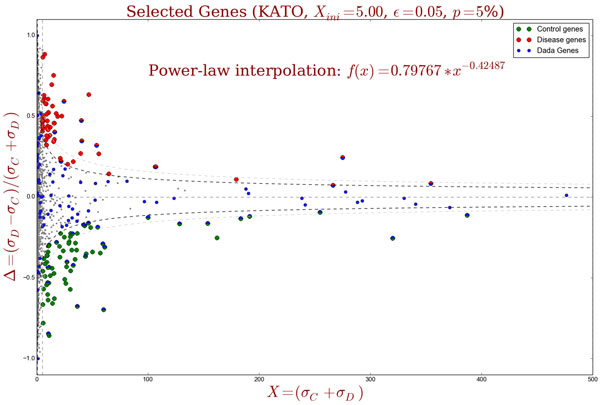
**Selection of top 5% genes by Δ*' *and DADA**. The Δ*' *score selects genes with largest alterations between *σ_C _*and *σ_D_*, after applying parametric interpolation by power-law. Red and green dots indicate genes with larger coexpression in disease and control, respectively. Gray dots represent genes which are not differentially altered between the two conditions. Blue dots indicate genes obtained by DADA. Genes with large *X *values participate in many selected shortest paths, which means that they have great importance with regard to the seeds, considering both topological centrality and expression concordance.

Figure 2 illustrates the top 5% genes selected by Δ*' *(red and green dots, indicating genes more coexpressed in disease and control, respectively). Gray dots represent genes which are not altered between the two conditions, while blue dots represent genes obtained by DADA (considering the same number of genes).

There exist some important differences between DADA and Δ*' *methods. In one hand, DADA aims to alleviate the ascertainment bias, but it does not worry about the differential alteration between two conditions. That is the reason by which it retrieves some genes that are not differentially altered (represented by gray dots in Figure 2). On the other hand, the score Δ*' *successfully retrieves differentially coexpressed genes and, at the same time, alleviates the ascertainment bias, since several genes with large *X *were not selected. In this way, by integrating different sources of biological data, our method can provide more specific results according to the differential coexpression of the genes (under two conditions).

#### Intersections and correlations of the rankings obtained by DADA, RWR, *X* and Δ*'*

**Figure 3:**
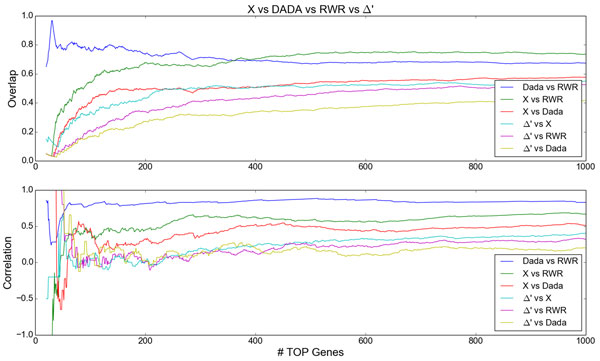
**Comparison of the methods DADA, RWR, *X *and Δ*'*, varying the number of top genes considered by each method**. **Top**: intersections (in proportion) between list pairs of top *N *genes obtained by each pair of methods. **Bottom**: Spearman correlations between list pairs of top *N *genes obtained by each pair of methods.

To compare the intersections and corrrelations of the rankings obtained by DADA, RWR, *X *and Δ*'*, first we select the list of top *N *genes from each method. Then, we extract the genes belonging to the intersection of the lists and evaluate the Spearman rank correlation of the intersection. Finally, this process is repeated for several values of *N*, resulting in curves of intersection and correlation along the *N *domain. Figure 3 presents the comparison of the intersection of the top *N *genes lists obtained by each pair of methods, varying *N *from 20 to 1000. In the top, curves of intersection (in proportion) between the top *N *genes obtained by each pair of methods are presented, while in the bottom, curves of Spearman correlation between the top *N *genes obtained by each pair of methods are presented.

We observe that, for a small number of top genes considered (*N *≤ 100), the intersection between DADA and RWR lists is remarkedly high (about 0.8), while the intersections between the remaining pairs of methods start relatively small and gradually increases with *N*. For the same considered domain (*N *≤ 100), intense fluctuations in correlations of genes belonging to the intersections between the pairs of methods can be observed. This is expected, since for *N *≤ 50, most of the intersections (except for DADA vs RWR) are very low, containing at most 3 genes, for which the correlations are not meaningful. Thus, small lists are prone to large correlation fluctuations. These fluctuations decrease as the intersection lists increase. For *N *≥ 100, all curves for both intersection and correlation tend to stabilize. Although the intersection between DADA and RWR is overcome by the intersection between *X *and RWR for *X *≥ 360, we notice that the correlation between DADA and RWR remains the largest (about 0.84). This is expected since DADA performs a statistical adjustment aiming to insert some genes with relatively small degree to the resulting list from RWR. In this way, the intersection between DADA and RWR is high in the beginning and tends to decrease as *N *increases. In its turn, the correlation between DADA and RWR is high in the beginning (*N *≥ 30), followed by a decrease (*N *∈ [31,38]) due to the insertion of genes resulting from DADA adjustment. After that, the correlation tends to increase until converging to approximately 0.84.

Additionally, the smallest intersections and correlations involved Δ*'*, which is expected since Δ*' *was idealized to capture alterations between control and disease. The largest intersection involving Δ*' *was with *X *(about 0.53) and the least one was with DADA (about 0.38). From *N *= 400 on, correlation between Δ*' *and all methods were small: about 0.1 with DADA, about 0.2 with RWR, and about 0.35 with *X*. This suggests that Δ*' *tends to capture novel genes (differentially co-expressed in both conditions) that are not obtained by other methods. Regarding *X*, even though it considers coexpression and location with regard to the seeds as the main factors, these are not considered in a differential way, resulting in moderated correlations with DADA and RWR.

#### Intersections and correlations of Degree with (DADA, RWR, *X*, Δ*'*)

**Figure 4:**
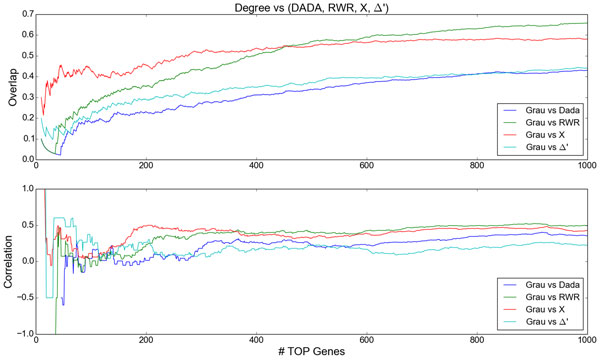
**Comparison of the genes ranking obtained by Degree regarding to the rankings obtained by DADA, RWR, *X *and Δ*'***. The number of top genes considered were varied from 20 to 1000. **Top**: intersections (in proportion) between the lists of the top genes obtained by degree with regard to DADA, RWR, *X *and Δ*'*. **Bottom**: correlations between the lists of the top genes obtained by degree with regard to other methods.

As discussed before, genes/proteins widely studied in the literature generally tend to have more interactions with its neighbors, thus presenting large degree. Recalling that this distortion observed in the degree distribution of PPI networks is known as ascertainment bias. Due to this fact, it is possible that genes associated with a given disease which were not well studied might be neglected by existing methods for gene priorization. Thus, in this part we analyze the intersections and correlations between the ranking ordered by Degree with regard to the rankings obtained by the four methods considered (DADA, RWR, *X*, Δ*'*). Figure 4 presents a comparison of the Degree ranking with the rankings obtained by DADA, RWR, *X *and Δ*'*, varying the number (*N *) of top genes considered.

We observe that, for a small number of top genes considered (*N *≤ 100), the intersection between Degree and *X *is the largest (varying between 0.21 and 0.46), while the intersections between Degree and other methods begin close to zero and gradually increase with *N *until reach the values 0.28, 0.21 and 0.19, respectively for RWR, Δ*' *and DADA. In the same interval, great fluctuations in the correlations of the Degree ranking with method rankings, due to the fact most of intersections are relatively small. For *N *≥ 200, the correlation curves begin to stabilize presenting less fluctuations. It is also possible to notice that the intersection between Degree and *X *is overcome by intersection between degree and RWR from *N *= 450, which shows that RWR tends to incorporate into its list a larger proportion of genes with large degree as *N *increases. The rankings achieved by Δ*' *and DADA present relatively small values of intersection and correlation with Degree along the whole domain. For *N *≥ 200, although the intersection between Δ*' *and Degree is slightly larger than the intersection between DADA and Degree, the correlation between Δ*' *and Degree present values slightly less or equal to the correlation between DADA and Degree.

At this point, we recall that the score *X *aims to find genes coexpressed in the neighborhood of the seeds in one or both conditions (control and disease). In the proposed method, even though the selection the shortest paths depends in great part on coexpression of the genes with the seed, genes with high degree and highly coexpressed tend to be prioritized by *X*, since such genes usually participate in many shortest paths between the seeds. Thus, *X *tends to achieve highly coexpressed and connected genes in the neighborhood of the seeds, which suggests that *X *is appropriate to find *PARTY HUBS *(proteins highly co-expressed with their partners, acting as local coordinators) [45]. For example, by fixing *N *= 100 and comparing genes belonging to the intersections (*Degree *∩ *X*) and (*Degree *∩ *RWR*), we observe that 27 out of 28 genes belonging to (*Degree *∩ *RWR*) also belong to (*Degree *∩ *X*), but (*Degree *∩ *X*) possesses 43 genes from which 16 genes do not belong to (*Degree *∩ *RWR*). However, we found that most of these 16 genes (NR3C1, GABARAPL1, UBC, TRAF6, APP, YWHAQ, EPB41, CTNNB1, MAPK1, IKBKG, PIK3R1, IKBKE, HSP90AA1, MDM2, RELA and ATF2.) are strongly related to schizophrenia [46-50]. This indicates that *X *captures more high degree genes related to the disease than RWR does. Another aspect to highlight regarding such intersections is that from *N *= 480 on, the intersection between *x *and Degree stabilizes, while RWR continues to present increasing intersection with Degree. This indicates that RWR is more prone to ascertainment bias.

Regarding the intersections of Δ*' *and DADA with Degree, they were smaller, which suggests that these scores are less prone to the ascertainment bias. Besides, it is important to highlight that the ranking achieved by DADA is strongly based on RWR ranking, except by the fact that the first tries to prioritize small degree genes in certain conditions. Recalling that Δ*' *seeks to prioritize the coexpression differences (with the seeds) of the genes in the selected shortest paths under two conditions (control and disease). Therefore, Δ*' *is more adequate to discover novel genes associated with a given disease.

#### Replication of KATO, ALTARC and BAHN studies

**Figure 5:**
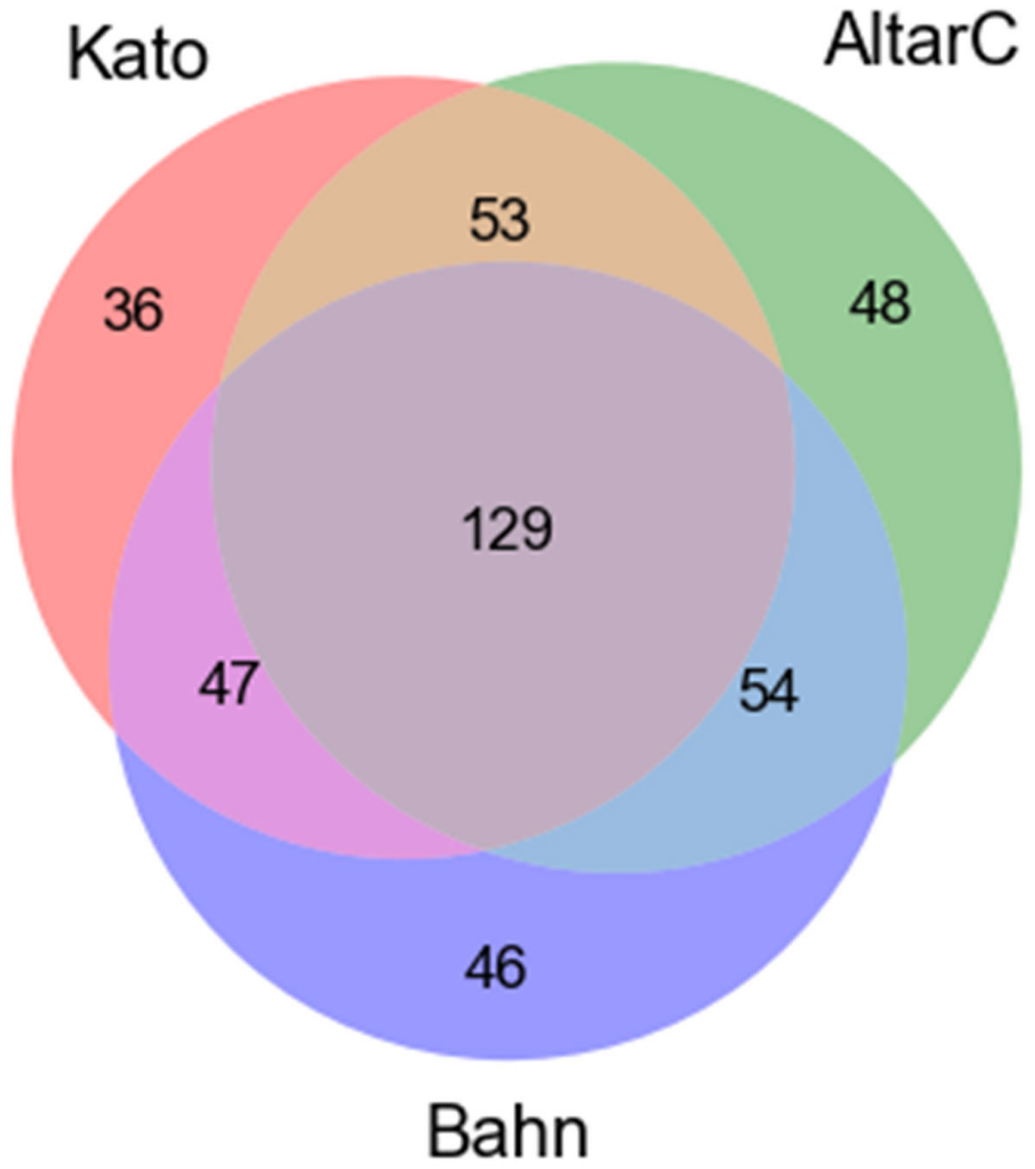
**Intersection of the genes ranked by Δ*'***. The top 10% of the genes ranked by Δ*' *were selected for each study: KATO, ALTARC and BAHN, resulting in an overlap of 129 genes (~46.9%).

We performed an analysis of replication (overlap) of the top genes ranked by both scores Δ*' *and *X *for KATO, ALTARC and BAHN studies. Figure 5 presents the intersections of the three lists with top 10% of the genes ranked by Δ*'*, one list per study. The number of genes obtained for KATO, ALTARC and BAHN studies were 265, 285 and 276, respectively.

Note that the intersection among the three lists contains 129 genes, which represents approximately 46.9% of the average number of genes per list. This number is remarkable, since the number of genes expected if the lists were obtained at random would be about 3, which correponds to about 0.1% (10% × 10% × 10%) of the average number of genes per study, since the three lists have approximately the same size. It is important to highlight that the genes belonging to this intersection are those which present desirable topological and coexpression characteristics (including differential co-expression between control and disease cases), not only for one, but for all three studies. A biological analysis of this list of 129 genes is presented in the next section.

**Figure 6:**
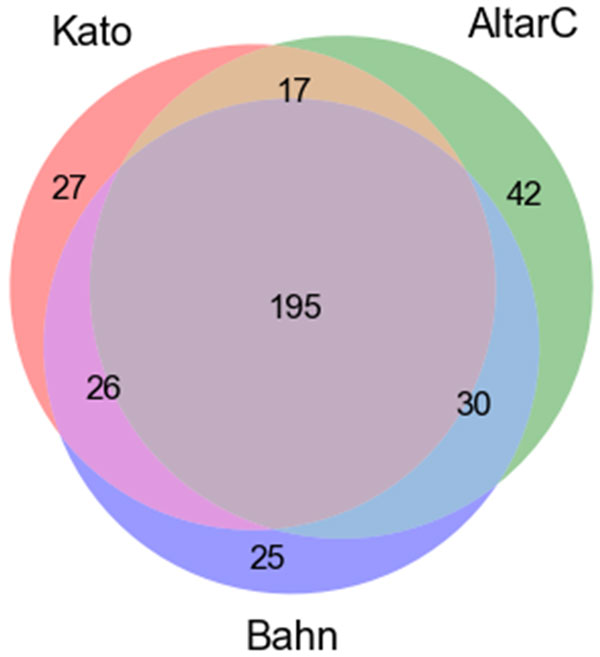
**Intersection of the genes ranked by *X***. The top 10% of the genes ranked by *X *were selected for each study: KATO, ALTARC and BAHN, resulting in an overlap of 195 genes (~70.9%).

Regarding the replication obtained by score *X*, Figure 6 presents the intersections of the three lists with top 10% of the genes, one list per study (KATO, ALTARC and BAHN). The intersection among the three lists resulted in 195 genes, which represents approximately 70.9% of the average number of genes per study. Thus, the rankings obtained by *X *presented even larger intersection than the obtained by Δ*' *rankings. This is expected, since genes highly coexpressed with the seed and, at the same time, highly central with regard to the seeds in both control and disease conditions tend to be prioritized by *X*. This is an evidence that *X *tends to recover genes widely studied in the literature and, at the same time, highly important with regard to the seeds considering both topological and coexpression aspects. Besides, many genes belonging to this intersection have potential to be *PARTY HUBS*. Biological analyses showed results similar to the analyses performed by rankings obtained by Δ*' *(biological analyses for Δ*' *are presented in the next section).

### Biological analyses

For the biological analyses, we submitted the considered gene lists to GO and KEGG analyses available in WebGestalt (WEB-based GEne SeT AnaLysis Toolkit - http://bioinfo.vanderbilt.edu/webgestalt/). In this way, we performed functional, hyper-represented pathways, and disease association analyses of the obtained genes.

#### List of top 10% genes ranked by Δ*' *for the KATO study

First, we analyzed the top 10% (265) genes ranked by Δ*' *for the KATO study. By performing GO analysis over this list of genes, searching the top 10 over represented biological functions with more than 10 genes per function and adjP < 0.05 (adjP: p-value adjusted by the multiple test adjustment). We observed the following functions as over represented: regulation of signal transduction (adjP = 1.95e-24); nerve growth factor receptor signaling pathway (adjP = 3.77e-24); intracellular protein kinase cascade (adjP = 2.07e-25); protein phosphorilation (adjP = 3.77e-24).

Using the same parameters described above on the KEGG analysis the important over represented pathways were: neurotrophin signaling pathways (adjP = 1.85e-33); pathways in cancer (adjP = 2.55e-32); MAPK signaling pathway (adjP = 2.7e-31); focal adhesion (adjP = 3.60e-28) and ErbB signaling pathway (adjP = 8.66e-25) - (see supplementary Table S1). We also performed an over represented analysis of diseases. It is noteworthy that cancer, stress, skin disorders and different psychiatry disorders were over represented (see supplementary Table S2). The aforementioned analyses were also performed using the top 10% genes ranked by score *X*, and the results were similar, regarding aspects of biological pathways (results not shown).

#### Intersection among 3 studies - ranked by Δ*'*

**Figure 7:**
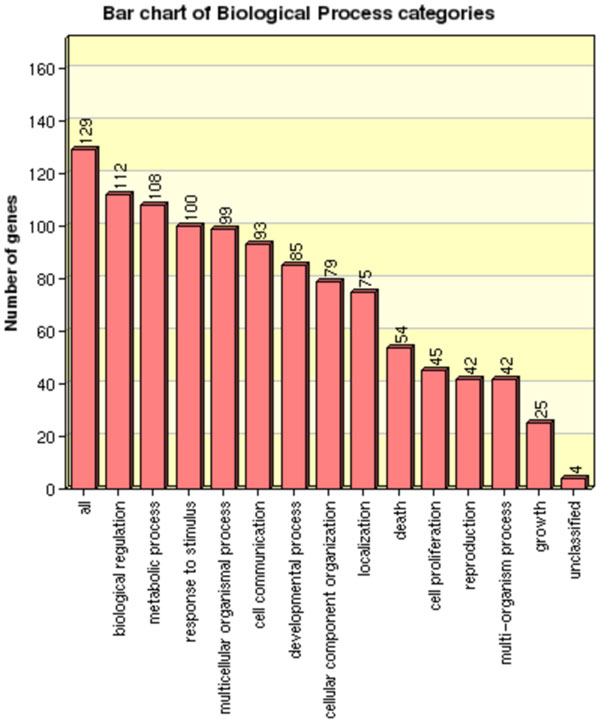
**Biological Process categories**. Distribution of the genes present in the intersection list obtained by Δ*' *top 10% rankings for KATO, ALTARC and BAHN studies. Some processes are associated to schizophrenia: cell communication, cell death (apoptosis), biological regulation, stimuli response and cell proliferation (neurogenesis ability).

Here we analyze the genes present in the intersection among the Δ*' *top 10% rankings obtained from KATO, ALTARC and BAHN studies. The Δ*' *intersection list presents 129 genes, and the distribution of these genes on their respective biological processes categories is presented in Figure 7. Among the biological processes related to schizophrenia [38,51,52], we highlight: biological regulation (112 genes), stimuli response (100 genes), cell communication (93 genes), developmental process (85 genes), apoptosis (54 genes) and cell proliferation (45 genes). This preliminary analysis indicates that these genes actually participate in biological processess related to schizophrenia, indicating that the intersection of the top genes ranked by Δ*' *- for the three considered studies - presents genes relevant to the disease in question.

In addition, we performed in these 129 genes an enrichment analysis representing the most important genes related to schizophrenia. As expected, the biological process and KEGG enrichment analysis (with more than 10 genes in each category and an adjusted P-value < 0.05) were similar to our previous analysis, since all 129 genes in this list are contained in the list of 265 genes obtained from KATO study. However, it is noteworthy that, in the first analysis, nothing specific related to neurons appear with such stringent criteria, but using the 129 genes (from intersection of 3 studies) in the cellular component enrichment analysis, neuron projections (adjP = 8.3e-19) were over represented. Another important point is that the disease enrichment analysis from the 129 genes also presented cancer, stress, neurodegenerative disorders and other psychiatric disorders.

**Figure 8:**
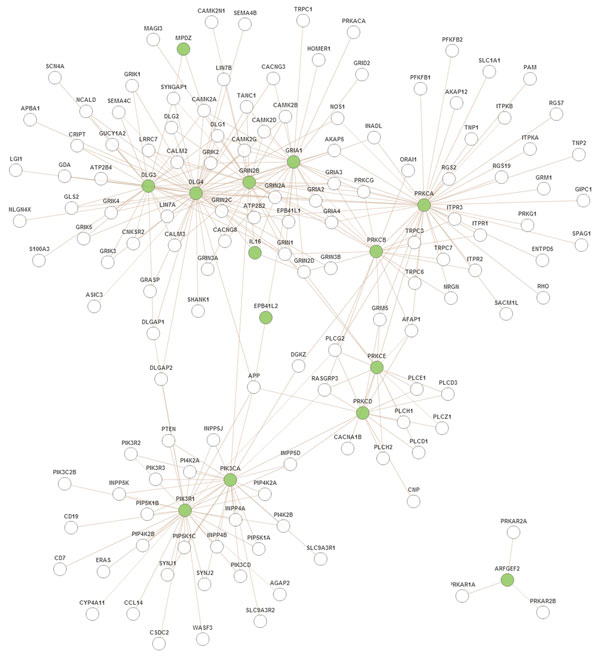
**PPI human module**. Module 26 with 14 genes (green nodes) belonging to the 129 overlapped genes from 3 studies. This module is related to glutamate receptor signaling pathway, a very important pathway related to schizophrenia.

Although other psychiatric disorders such as depression, and Alzheimer also appeared over represented besides schizophrenia, another interesting point is that, considering the same criteria in both analysis, the majority of psychiatric disorders that appear over represented in the KATO analysis (such as Asperger, panic disorder and eating disorder) didn't appear with the 129 genes from intersection list (see supplementary Table S3). We searched for PPI human modules over represented in the 129 genes and we found 5 PPI Modules (see supplementary Figure S4). The most interesting module was Module 26 (adjp = 0.0021) with 14 genes represented in our list and just found in the 129 gene set. This module is related to glutamate receptor signaling pathway, a very important pathway related to schizophrenia. Figure 8 represents the PPI Module 26 from Human Interactome (WebGestalt tools). Green nodes represent genes from our 129 gene set.

We conclude with our biological analysis that our method is able to select genes already related to schizophrenia, as well as point new genes and pathways that can provide good candidates. Also we observe that our method achieves a great overlap (46,9%) among different studies that, by using only conventional methods which perform differential expression analyses, achieved almost null intersection among the resulting lists. In addition to achieve a concordance among studies, the method was also able to select a more restricted set of genes related to a specific disorder.

## Conclusion

In this work, we presented an integrative approach combining data from gene expression, PPI network and GWAS to prioritize genes potentially related to complex diseases. By assuming some network medicine hypothesis, the method explores the neighborhood of a gene set by locating paths possessing more coexpressed genes with seeds - this is independently performed for two conditions (control and disease). Our method outputs two scores *X *and Δ*'*. The first one (*X*) prioritizes genes with *party hub *features, possessing high topological centrality and, at the same time, high coexpression relative to the seed genes. The second one (Δ*'*) prioritizes the most altered genes between two conditions. We performed a comparative analysis involving our method and two state-of-art methods (RWR and DADA) by using schizophrenia as case study. Results showed that our method (both scores *X *and Δ*'*) complements RWR and DADA by obtaining genes which present a good balance between topological centrality and differential gene coexpression. Additionally, similarly to DADA, the score Δ*' *does not present the ascertainment bias problem. However, our method does not require parameters adjustment and, at the same time, achieves a great replicability in the selection of important genes from different microarray studies of the same disease, producing a more specific gene set related to the studied disorder. Besides, the score Δ*' *prioritized genes belonging to biological pathways highly related to schizophrenia, indicating that this score could be used for gene discovering. On the other hand, the score *X *prioritized genes related to schizophrenia that are highly referred by literature. Therefore, by integrating gene coexpression with PPI network, our method achieves more specific and restrictive results which can allow gene discovering. Besides, both methods presented high replicability results among three different microarray studies

## Competing interests

The authors declare that they have no competing interests.

## Authors' contributions

SNS, DCMJ, HB and RFH conceived and designed the study. SNS and DCMJ participated in reviewing the literature, designed and drafted the manuscript. CABP contributed with statistical concepts about the criterion for shortest paths selection (modified Kendall concordance of gene expressions). SNS worked on the implementation of the methodology. SNS and HB performed the biological analysis. DCMJ, RFH and HB guided throughout the whole study, including conceptualization, designing, application, and editing the manuscript. All authors revised and approved the manuscript.

## Declarations

We would like to thank IFES, FAPESP grants # 2010/52138-8 and # 2011/50761-2, CNPq, CAPES, NAP eScience PRP - USP for financial support. The publication charges for this article were funded by FAPESP grant # 2011/50761-2.

This article has been published as part of *BMC Bioinformatics *Volume 16 Supplement 19, 2015: Brazilian Symposium on Bioinformatics 2014. The full contents of the supplement are available online at http://www.biomedcentral.com/bmcbioinformatics/supplements/16/S19

## References

[B1] Schadt EE Molecular networks as sensors and drivers of common human diseases Nature 2009 461 7261 218 23 doi:10.1038/nature08454 19741703 10.1038/nature08454

[B2] McCarthy MI Abecasis GR Cardon LR Goldstein DB Little J Ioannidis JPa Hirschhorn JN Genome-wide association studies for complex traits: consensus, uncertainty and challenges Nature reviews Genetics 2008 9 5 356 69 doi:10.1038/nrg2344 10.1038/nrg2344 18398418

[B3] Wang K Li M Hakonarson H Analysing biological pathways in genome-wide association studies Nature reviews. Genetics 2010 11 12 843 54 doi:10.1038/nrg2884 10.1038/nrg2884 21085203

[B4] Solovieff N Cotsapas C Lee PH Purcell SM Smoller JW Pleiotropy in complex traits: challenges and strategies Nature reviews. Genetics 2013 14 7 483 95 doi:10.1038/nrg3461 10.1038/nrg3461 PMC4104202 23752797

[B5] Li L Kabesch M Bouzigon E Demenais F Farrall M Moffatt MF Lin X Liang L Using eQTL weights to improve power for genome-wide association studies: a genetic study of childhood asthma Frontiers in genetics 2013 4 May 103 doi:10.3389/fgene.2013.00103 23755072 10.3389/fgene.2013.00103 PMC3668139

[B6] Nica AC Dermitzakis ET Expression quantitative trait loci: present and future Philosophical transactions of the Royal Society of London. Series B, Biological sciences 2013 368 1620 20120362 doi:10.1098/rstb.2012.0362 23650636 10.1098/rstb.2012.0362 PMC3682727

[B7] Barabási A-l Gulbahce N Loscalzo J Network medicine: a network-based approach to human disease Nature reviews. Genetics 2011 12 1 56 68 doi:10.1038/nrg2918 10.1038/nrg2918 PMC3140052 21164525

[B8] Kohler S Bauer S Horn D Robinson PN Ko S Walking the Interactome for Prioritization of Candidate Disease Genes Journal of Human Genetics 2008 82 April 949 958 doi:10.1016/j.ajhg.2008.02.013 10.1016/j.ajhg.2008.02.013 PMC2427257 18371930

[B9] Chen J Aronow BJ Jegga AG Disease candidate gene identification and prioritization using protein interaction networks BMC bioinformatics 2009 10 73 doi:10.1186/1471-2105-10-73 19245720 10.1186/1471-2105-10-73 PMC2657789

[B10] Vanunu O Magger O Ruppin E Shlomi T Sharan R Associating genes and protein complexes with disease via network propagation PLoS computational biology 2010 6 1 100064 doi:10.1371/journal.pcbi.1000641 10.1371/journal.pcbi.1000641 PMC2797085 20090828

[B11] Wu X Jiang R Zhang MQ Li S Network-based global inference of human disease genes Molecular systems biology 2008 4 189 189 doi:10.1038/msb.2008.27 18463613 10.1038/msb.2008.27 PMC2424293

[B12] Tong H Faloutsos C Pan J-Y Random walk with restart: fast solutions and applications Knowledge and Information Systems 2007 14 3 327 346 doi:10.1007/s10115-007-0094-2

[B13] Edwards AM Kus B Jansen R Greenbaum D Greenblatt J Gerstein M Bridging structural biology and genomics: assessing protein interaction data with known complexes Drug discovery today 2004 9 2 Suppl 32 40 23573642

[B14] Hart GT Ramani AK Marcotte EM How complete are current yeast and human protein-interaction networks? Genome biology 2006 7 11 120 doi:10.1186/gb-2006-7-11-120 17147767 10.1186/gb-2006-7-11-120 PMC1794583

[B15] Erten S Bebek G Ewing RM Koyutürk M DADA: Degree-Aware Algorithms for Network-Based Disease Gene Prioritization BioData mining 2011 4 1 19 doi:10.1186/1756-0381-4-19 21699738 10.1186/1756-0381-4-19 PMC3143097

[B16] Kim Y-a Wuchty S Przytycka TM Identifying causal genes and dysregulated pathways in complex diseases PLoS computational biology 2011 7 3 1001095 doi:10.1371/journal.pcbi.1001095 10.1371/journal.pcbi.1001095 PMC3048384 21390271

[B17] Ulitsky I Karp R Detecting disease-specific dysregulated pathways via analysis of clinical expression profiles Research in Computational Molecular Biology 2008 4955 347 359

[B18] Franke L van Bakel H Fokkens L de Jong ED Egmont-Petersen M Wijmenga C Reconstruction of a functional human gene network, with an application for prioritizing positional candidate genes American journal of human genetics 2006 78 6 1011 25 doi:10.1086/504300 16685651 10.1086/504300 PMC1474084

[B19] Suthram S Beyer A Karp RM Eldar Y Ideker T eQED: an efficient method for interpreting eQTL associations using protein networks Molecular systems biology 2008 4 162 162 doi:10.1038/msb.2008.4 18319721 10.1038/msb.2008.4 PMC2290938

[B20] Keller A Backes C Gerasch A Kaufmann M Kohlbacher O Meese E Lenhof H-P A novel algorithm for detecting differentially regulated paths based on gene set enrichment analysis Bioinformatics (Oxford, England) 2009 25 21 2787 94 doi:10.1093/bioinformatics/btp510 10.1093/bioinformatics/btp510 PMC2781748 19713416

[B21] Mitra K Carvunis A-R Ramesh SK Ideker T Integrative approaches for finding modular structure in biological networks Nature reviews. Genetics 2013 14 10 719 32 doi:10.1038/nrg3552 10.1038/nrg3552 PMC3940161 24045689

[B22] Xu L-m Li J-R Huang Y Zhao M Tang X Wei L AutismKB: an evidence-based knowledgebase of autism genetics Nucleic acids research 2012 40 Database 1016 22 doi:10.1093/nar/gkr1145 10.1093/nar/gkr1145 PMC3245106 22139918

[B23] Basu SN Kollu R Banerjee-Basu S AutDB: a gene reference resource for autism research Nucleic acids research 2009 37 Database 832 6 doi:10.1093/nar/gkn835 19015121 10.1093/nar/gkn835 PMC2686502

[B24] Kim S Webster MJ The stanley neuropathology consortium integrative database: a novel, web-based tool for exploring neuropathological markers in psychiatric disorders and the biological processes associated with abnormalities of those markers Neuropsychopharmacology : official publication of the American College of Neuropsychopharmacology 2010 35 2 473 82 doi:10.1038/npp.2009.151 19829293 10.1038/npp.2009.151 PMC3055386

[B25] Higgs BW Elashoff M Richman S Barci B An online database for brain disease research BMC genomics 2006 7 70 doi:10.1186/1471-2164-7-70 16594998 10.1186/1471-2164-7-70 PMC1489945

[B26] Sullivan PPF Kendler KS Neale MC Schizophrenia as a complex trait: evidence from a meta-analysis of twin studies Archives of general psychiatry 2003 60 12 1187 92 doi:10.1001/archpsyc.60.12.1187 14662550 10.1001/archpsyc.60.12.1187

[B27] Jia P Sun J Guo aY Zhao Z SZGR: a comprehensive schizophrenia gene resource Molecular psychiatry 2010 15 5 453 62 doi:10.1038/mp.2009.93 20424623 10.1038/mp.2009.93 PMC2861797

[B28] Prasad TSK Goel R Kandasamy K Keerthikumar S Kumar S Mathivanan S Telikicherla D Raju R Shafreen B Venugopal A Keshava Prasad TS Balakrishnan L Marimuthu A Banerjee S Somanathan DS Sebastian A Rani S Ray S Harrys Kishore CJ Kanth S Ahmed M Kashyap MK Mohmood R Ramachandra YL Krishna V Rahiman BA Mohan S Ranganathan P Ramabadran S Chaerkady R Pandey A Human Protein Reference Database - 2009 update Nucleic Acids Research 2009 37 Database issue 767 772 doi:10.1093/nar/gkn892 10.1093/nar/gkn892 PMC2686490 18988627

[B29] Kerrien S Aranda B Breuza L Bridge A Broackes-Carter F Chen C Duesbury M Dumousseau M Feuermann M Hinz U Jandrasits C Jimenez RC Khadake J Mahadevan U Masson P Pedruzzi I Pfeiffenberger E Porras P Raghunath A Roechert B Orchard S Hermjakob H The IntAct molecular interaction database in 2012 Nucleic acids research 2012 40 Database issue 841 6 doi:10.1093/nar/gkr1088 10.1093/nar/gkr1088 PMC3245075 22121220

[B30] Licata L Briganti L Peluso D Perfetto L Iannuccelli M Galeota E Sacco F Palma A Nardozza AP Santonico E Castagnoli L Cesareni G MINT, the molecular interaction database: 2012 update Nucleic acids research 2012 40 Database 857 61 doi:10.1093/nar/gkr930 10.1093/nar/gkr930 PMC3244991 22096227

[B31] Bolstad BM Irizarry RA Astrand M Speed TP A comparison of normalization methods for high density oligonucleotide array data based on variance and bias Bioinformatics (Oxford, England) 2003 19 2 185 93 10.1093/bioinformatics/19.2.185 12538238

[B32] Irizarry Ra Hobbs B Collin F Beazer-Barclay YD Antonellis KJ Scherf U Speed TP Exploration, normalization, and summaries of high density oligonucleotide array probe level data Biostatistics (Oxford, England) 2003 4 2 249 64 doi:10.1093/biostatistics/4.2.249 10.1093/biostatistics/4.2.249 12925520

[B33] Aittokallio T Schwikowski B Graph-based methods for analysing networks in cell biology Briefings in bioinformatics 2006 7 3 243 55 doi:10.1093/bib/bbl022 16880171 10.1093/bib/bbl022

[B34] Chuang JS Roth D Gene recognition based on DAG shortest paths Bioinformatics (Oxford, England) 2001 17 Suppl 1 56 64 10.1093/bioinformatics/17.suppl_1.s56 11472993

[B35] Managbanag JR Witten TM Bonchev D Fox La Tsuchiya M Kennedy BK Kaeberlein M Shortest-path network analysis is a useful approach toward identifying genetic determinants of longevity PloS one 2008 3 11 3802 doi:10.1371/journal.pone.0003802 10.1371/journal.pone.0003802 PMC2583956 19030232

[B36] Missiuro PV Liu K Zou L Ross BC Zhao G Liu JS Ge H Information flow analysis of interactome networks PLoS computational biology 2009 5 4 1000350 doi:10.1371/journal.pcbi.1000350 10.1371/journal.pcbi.1000350 PMC2685719 19503817

[B37] Przulj N Wigle Da Jurisica I Functional topology in a network of protein interactions Bioinformatics (Oxford, England) 2004 20 3 340 8 doi:10.1093/bioinformatics/btg415 10.1093/bioinformatics/btg415 14960460

[B38] Sun J Jia P Fanous AH van den Oord E Chen X Riley BP Amdur RL Kendler KS Zhao Z Schizophrenia gene networks and pathways and their applications for novel candidate gene selection PloS one 2010 5 6 11351 doi:10.1371/journal.pone.0011351 10.1371/journal.pone.0011351 PMC2894047 20613869

[B39] Stuart JM Segal E Koller D Kim SK A gene-coexpression network for global discovery of conserved genetic modules Science (New York, N.Y.) 2003 302 5643 249 55 doi:10.1126/science.1087447 10.1126/science.1087447 12934013

[B40] Kendall MG Smith BB The Problem of $m$ Rankings The Annals of Mathematical Statistics 1939 10 3 275 287 doi:10.1214/aoms/1177732186

[B41] Kumari S Nie J Chen H-S Ma H Stewart R Li X Lu M-Z Taylor WM Wei H Evaluation of gene association methods for coexpression network construction and biological knowledge discovery PloS one 2012 7 11 50411 doi:10.1371/journal.pone.0050411 10.1371/journal.pone.0050411 PMC3511551 23226279

[B42] White S Smyth P Algorithms for estimating relative importance in networks Proceedings of the Ninth ACM SIGKDD International Conference on Knowledge Discovery and Data Mining - KDD '03 2003 ACM Press, New York, New York, USA 266 doi:10.1145/956755.956782

[B43] Hecker M Goertsches RH Engelmann R Thiesen H-J Guthke R Integrative modeling of transcriptional regulation in response to antirheumatic therapy BMC bioinformatics 2009 10 262 doi:10.1186/1471-2105-10-262 19703281 10.1186/1471-2105-10-262 PMC2757030

[B44] Dudoit S Yang YH Callow MJ Speed TP Statistical methods for identifying differentially expressed genes in replicated cDNA microarray experiments Statistica sinica 2002 12 3 111 139

[B45] Agarwal S Deane CM Porter MA Jones NS Revisiting date and party hubs: Novel approaches to role assignment in protein interaction networks PLoS Comput Biol 2010 6 6 1000817 10.1371/journal.pcbi.1000817 PMC2887459 20585543

[B46] Sinclair D Webster M Fullerton J Weickert C Glucocorticoid receptor mrna and protein isoform alterations in the orbitofrontal cortex in schizophrenia and bipolar disorder BMC Psychiatry 2012 12 1 84 22812453 10.1186/1471-244X-12-84 PMC3496870

[B47] Hashimoto R Y Y Ohi K Variants of the rela gene are associated with schizophrenia and their startle responses Neuropsychopharmacology 2011 36 9 1921 1931 21593732 10.1038/npp.2011.78 PMC3154111

[B48] Kido M Nakamura Y Nemoto K Takahashi T Aleksic B Furuichi A Nakamura Y Ikeda M Noguchi K Kaibuchi K Iwata N Ozaki N Suzuki M The polymorphism of ywhae, a gene encoding 14-3-3epsilon, and brain morphology in schizophrenia: A voxel-based morphometric study PLoS ONE 2014 9 8 103571 10.1371/journal.pone.0103571 PMC4126687 25105667

[B49] Pandey G Rizavi H Tripathi M Ren X Region-specific dysregulation of glycogen synthase kinase-3*β *and *β*-catenin in the postmortem brains of subjects with bipolar disorder and schizophrenia Bipolar Disord 2014 in press 10.1111/bdi.12228 PMC4287464 25041379

[B50] Sinclair D Fillman S Webster M Weickert C Dysregulation of glucocorticoid receptor co-factors fkbp5, bag1 and ptges3 in prefrontal cortex in psychotic illness Sci Rep 2013 3 3539 24345775 10.1038/srep03539 PMC3866598

[B51] Fan Y Abrahamsen G McGrath JJ Mackay-Sim A Altered cell cycle dynamics in schizophrenia Biological psychiatry 2012 71 2 129 35 doi:10.1016/j.biopsych.2011.10.004 22074612 10.1016/j.biopsych.2011.10.004

[B52] Beaulieu J-M Sotnikova TD Marion S Lefkowitz RJ Gainetdinov RR Caron MG An Akt/beta-arrestin 2/PP2A signaling complex mediates dopaminergic neurotransmission and behavior Cell 2005 122 2 261 273 16051150 10.1016/j.cell.2005.05.012

